# Increased expression of interleukin-22 and its receptor is relevant to poor prognosis in laryngeal squamous cell carcinoma: A case control trial

**DOI:** 10.1097/MD.0000000000028419

**Published:** 2021-12-23

**Authors:** Wenjun Ji, Jing Li, Xin Wang, Dongsheng Gao, Tiantian Zhang

**Affiliations:** Department of Otorhinolaryngology & Head and Neck Surgery, The People's Hospital of Su Zhou New District, Su Zhou, China.

**Keywords:** aryl hydrocarbon receptor, interleukin 22 receptor, interleukin-22, laryngeal squamous cell carcinoma, signal transducer and activator of transcription 3

## Abstract

To detect the expression of interlerukin-22 (IL-22) and associated genes and to evaluate their relationship with clinicopathological features and prognosis in laryngeal squamous cell carcinoma (LSCC).

The expression of IL-22 and associated genes were evaluated by immunohistochemistry and real time polymerase chain reaction in LSCC tissues from 30 patients and adjacent non-tumor tissues. A statistical analysis was implemented to assess the relationship among levels of expression, clinicopathological factors, and overall survival.

The expression of IL-22 and interleukin 22 receptor 1 (IL-22R1) was mainly located in the cytoplasm, and the expression of LSCC was significantly higher than in controls. The expression of aryl hydrocarbon receptor and signal transducer and activator of transcription 3 distributed in the cell nucleus, which was significantly higher in LSCC than in controls. The expression of IL-22 and IL-22R1 was associated with metastasis of lymph node and clinical stage of LSCC. Overall survival of LSCC was significantly poorer with higher expression of IL-22 and IL-22R1 than in those with lower expression.

The present research indicated that the increased level of IL-22 and IL-22R1 may be related to the pathogenesis and prognosis of LSCC. IL-22 may be the important biomarker, which need further research.

## 1 Introduction

Head and neck cancer is the sixth common malignancy worldwide and the seventh leading cause of death. Laryngeal cancer is one of the most common head and neck malignancy which seriously threaten physical and mental health of the patient. Laryngeal squamous cell carcinomas (LSCC) is the principal pathological type of laryngeal malignancy, the percentage is 85% to 90% approximately.^[1]^ There are 30% to 50% of all neoplasms in the head and neck are LSCC. In spite of the advances in the treatment, the mortality of LSCC is still high. Postoperative recurrence, metastasis, and insensitive to radiotherapy and chemotherapy often occur in patients with LSCC.^[2]^ Although a large number of researches have been done to investigate the pathogenesis and prognosis of LSCC, the precise mechanism of LSCC is still unknown.

Previous studies have shown that inflammation contributes to the proliferation, migration, and survival of cancer cells, which may lead to tumor invasion and metastasis.^[3]^ Interleukin (IL)-22 is a member of cytokines of the IL-10 family.^[4]^ The signaling of IL-22 receptor in epithelial cells promotes cell defense programs by partly regulating the proliferation and survival of cells. IL-22 receptor is a heterodimeric receptor which consisted of 2 chains, they are IL-22R1 and IL-22R2. The expression of IL-22R2 chain is ubiquitously, however, the expression of the IL-22R1 chain is restricted to epithelial cells and stroma cell elements. In addition, the expression of IL-22 is cooperatively regulated by the transcription factors, retinoic acid orphan receptor γt (RORγt), and aryl hydrocarbon receptor (AhR).^[5]^

The role of IL-22 in cancer is ambivalent. The impact of IL-22 in cancer is described as both promoting and restraining tumor growth. In addition, IL-22 have the diverse role in many types of tumors, such as the colon, lung, gastric, liver, pancreatic, breast, bladder, thyroid, brain cancer, and lymphoma.^[5]^ Previous study suggested that the deregulation of the IL-22/IL-22R1 signaling axis is relevant to the increase, susceptibility and poor prognosis in several cancers, no matter in human or mice.^[6]^ In human pancreatic ductal adenocarcinoma, it has been revealed that IL-22 and IL-22R not only play positive roles in tumor invasion and metastasis, but also predict a poor prognosis.^[7]^ Recent research indicated that the IL-22/IL-22R1 in breast cancer cells is associated with high tumor-associated macrophages (TAMs) infiltrating and poor prognosis.^[8]^ These suggested that IL-22 may be one of the targets for developing anti-cancer therapies. Generally, squamous cell carcinomas are surrounded by inflammatory cells. In addition, it has been reported that IL-22 affects several important signaling pathway of oral squamous cell carcinoma cells via the signal transducer and activator of transcription 3 (STAT3)-dependent and/or -independent pathways.^[9]^ However, there is little knowledge in the potential roles of IL-22 in LSCC.

In this study, we detected the expression of mRNA and protein of IL-22, IL-22R1, and relevant factors in LSCC, such as transcription factors, RORγt (encoded by RORc) and AhR, and STAT 3. We analyzed the regulatory role between these factors according to the expression, and the clinicopathological factors and prognosis of LSCC. We had investigated the function of IL-22 in the pathogenesis of LSCC and discussed its importance in clinical diagnosis and treatment.

## 2 Materials and methods

### 2.1 Patients and samples

All the samples were collected from the inpatients in the department of Otorhinolaryngology–Head and Neck surgery at the People's Hospital of Su Zhou New District from April 2013 to January 2014. The inclusion criteria are: all the selected cases were confirmed by pathological diagnosis and had the complete data of clinical history and follow-up for 5 years at least. LSCC tissues and adjacent control tissues were obtained from 30 patients after surgery. The exclusion criteria are: the patients had received adjuvant therapy such as chemotherapy, radiation therapy, or immunotherapy 2 months prior to surgery; patients with other tumors; patients with diseases of important organs. The histomorphology of LSCC specimens was confirmed by hematoxylin and eosin (H&E) staining by the Department of Pathology of hospital. Non-neoplastic tissues of the control group were collected from the mucosal of larynx and hypopharynx undergoing the total laryngectomy, which were confirmed by the histology. Clinical stages of tumors were classified accordance to UICC 2010 TNM standard.^[10]^ Stage 0: Tis N0 M0; Stage I: T1 N0 M0; Stage II: T2 N0 M0; Stage III: T3 N0 M0, T1 N1 MO, T2 N1 M0, T3 N1 M0; Stage IV A: T4a N0 M0, T4a N1 M0, T1 N2 M0, T2 N2 M0, T3 N2 M0, T4a N2 M0, Stage IV B: T4b any N M0, any T3 M0; Stage IV C: any T any N M1. Two tissue sections were collected from each patient. One was snap-frozen for RNA extraction, and the other was fixed in 10% formaldehyde solution for immunohistochemical staining. Overall survival was defined as the time elapsed from surgery to death from any cause. This study was approval from the Ethics Committee of the People's Hospital of Su Zhou New District. Written informed consent was obtained from all participants before surgery.

### 2.2 Immunohistochemistry

Immunohistochemical staining was performed on 5 μm paraffin sections. The sections were mounted and heated at 64 °C for 30 minutes. Serial sections from each block were deparaffinized and hydrated. The endogenous peroxide activity was eliminated by treatment with 3% hydrogen peroxide for 10 minutes. 10% goat serum was used to block the sections for 30 minutes. Each section was incubated by the primary antibodies respectively at 4 °C overnight. Primary antibodies were anti-IL-22 (1:300), anti-IL22R (1:300), anti-RorC (1:100), anti-AhR (1:50), and anti-STAT3 (1:30) (Abcam, HongKong). Negative controls utilized PBS instead of primary antibodies. The sections were incubated with species-matched secondary antibodies at 37 °C for 30 minutes. The slides were washed 3 times with PBS, and then visualized by diaminobenzidine. Following a final wash, the slides were mounted, cover slipped, and sealed.

The percentage of immunostaining-positive cells and intensity scores were calculated respectively. Ten HP fields per sample were randomly selected and scored by 2 independent observers under the microscope. The percentage of positive cells was classified as score 1: <25%; 2: 25% to 50%; 3: 50% to 75%; 4: 75% to 100%. The intensity score was calculated as 0: no staining; 1: weak staining; 2: moderate staining; 3: strong staining. Total immunostaining score was assessed as the intensity score multiply by the percentage of positive cells, which ranged from 0 to 12. We defined score ≤6 as low expression, while score >6 as high expression.

### 2.3 Real time polymerase chain reaction (PCR)

Total RNA was extracted with Trizol (Invitrogen, Carlsbad, CA) according to the manufacturer's instructions. Total RNA was incubated with oligo(dT) and reverse transcriptase for 1 hour at 42 °C. The enzyme was inactivated for 5 minutes at 95 °C. The cDNA was storing at −20 °C. Real time PCR amplification was carried out to detect the expression of mRNA. β-Actin was used as an internal control. Each sample was performed in duplicating. Reverse and forward primers were listed in Table 1. PCR was performed under the following conditions: initial denaturation at 95 °C for 30 seconds, then 40 cycles of 95 °C for 5 seconds, 55 °C for 5 seconds, and 72 °C for 30 seconds. The level of the relative expression of each targeted gene was normalized using the ΔΔCT comparative method, based on the reference gene threshold cycle (CT) values. Sequence of primers are showed in Table 1.

**Table 1:** Sequence of primers.

Primers	Sequence	
IL-22	Forward	5′-GCAGGCTTGACAAGTCCAACT-3′
	Reverse	5′-GCCTCCTTAGCCAGCATGAA-3′
IL-22R1	Forward	5′-GAAGTCCTGCAACCTGACG-3′
	Reverse	5′-GGTAGTGTGCTGCAGAGAGC-3′
AHR	Forward	5′-ATCACCTACGCCAGTCGCAAG -3′
	Reverse	5′-AGGCTAGCCAAACGGTCCAAC-3′
RORc	Forward	5′-AGTCGGAAGGCAAGATCAGA-3′
	Reverse	5′-CAAGAGAGGTTCTGGGCAAG-3′
β-Actin	Forward	5′-CGTTGACATCCGTAAAGACC-3′
	Reverse	5′-AACAGTCCGCCTAGAAGCAC-3′

IL-22 = interlerukin-22, IL-22R1 = interleukin 22 receptor 1.

### 2.4 Statistical analysis

The data were reported as mean ± SD. The statistical significance between groups was assessed by unpaired *t* test and Chi square (or Fisher exact) test. *P* < .05 was considered to indicate a statistically significant difference. The Kaplan–Meier method survival was used to assess the survival curves and the differences between curves were evaluated by log-rank test. Statistical analysis was performed with SPSS version 22.0 (IBM Inc, New York, USA).

## 3 Results

### 3.1 Characteristics of patient

Association between IL-22 and IL-22R expression and clinicopathologic characteristics are showed in Table 2. In general information, such as age, gender, and tumor location, there was no significant difference between the 2 groups. The expressions of IL-22 and IL-22L were significantly different in the cases of lymphatic metastasis and clinical stage.

**Table 2:** Association between IL-22 and IL-22R expression and clinicopathologic characteristics.

		IL-22			IL-22R		
Variables	N	Low	High	*P* value	Low	High	*P* value
Age, y							
≤60	23 (76.7%)	18 (78.3%)	5 (21.7%)	.153	20 (87.0%)	3 (13.0%)	.565
>60	7 (23.3%)	3 (42.9%)	4 (57.1)		5 (71.4%)	2 (28.6%)	
Gender							
Male	25 (83.3%)	18 (72.0%)	7 (28.0%)	.662	21 (84.0%)	4 (16.0%)	>.99
Female	5 (16.7)	3 (60.0%)	2 (40.0%)		4 (80.0%)	1 (20.0%)	
Tumor location							
Supraglottic	22 (73.3%)	14 (63.6%)	8 (36.4%)	.374	18 (81.8%)	4 (18.2%)	>.99
Glottic	8 (26.7%)	7 (87.5%)	1 (12.5%)		7 (87.5%)	1 (12.5%)	
T stage							
T1–T2	10 (33.3%)	9 (90.0%)	1 (10.0%)	.204	10 (100%)	0 (0.0%)	.140
T3–T4	20 (66.7%)	12 (60.0%)	8 (40.0%)		15 (75.0%)	5 (25.0%)	
Lymphatic metastasis							
N0	17 (56.7%)	16 (94.1%)	1 (5.9%)	.002^∗^	17 (100%)	0 (0.0)	.009^∗^
N+	13 (43.3%)	5 (38.5%)	8 (61.5%)		8 (61.5%)	5 (38.5%)	
Differentiation							
High	11 (36.7%)	11 (100%)	0 (0.0%)	.061	10 (90.9%)	1 (9.1%)	.626
Moderate-poor	19 (63.3%)	13 (68.4%)	6 (31.6%)		15 (78.9%)	4 (21.1%)	
Clinical stage							
I + II	5 (16.7%)	5 (100%)	0 (0.0%)	.001^∗^	5 (100%)	0 (0.0%)	.037^∗^
III	17 (56.7%)	14 (82.4%)	3 (17.6%)		16 (94.1%)	1 (5.9%)	
IV	8 (26.7%)	3 (37.5%)	5 (62.5%)		4 (50.0%)	4 (50.0%)	

Fisher exact test. IL-22 = interlerukin-22, IL-22R = interleukin 22 receptor.

∗ *P* < .05.

### 3.2 The protein expression in immunohistochemistry

**Figure 1:**
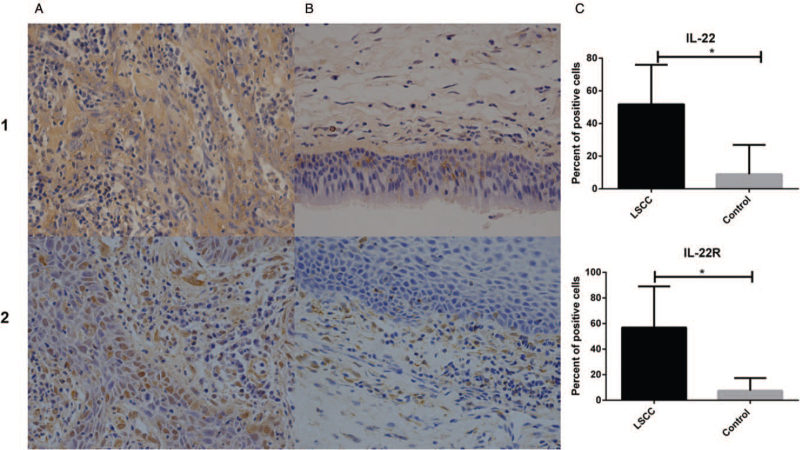
Immunohistochemical staining (A: LSCC; B: Controls) and the percentage of positive cells (C) of IL-22 (1) and IL-22R (2). Images acquired by optical microscope 400×. ^∗^*P* < .01; IL-22 = interlerukin-22, IL-22R = interleukin 22 receptor, LSCC = laryngeal squamous cell carcinoma.

Immunohistochemical staining showed that IL-22 protein was mainly distributed in the cytoplasm, but rarely in the cell nucleus. In the control tissues, the expression of IL-22 was predominantly located in the squamous epithelial cells and inflammatory cells, especially CD4+ T cells and macrophages. IL-22 was positive in tumor cells, mesenchyme and fibro tissues around cancer nests of LSCC. The protein expression of IL-22 in LSCC was significantly higher than in controls (*P* = .001) (shown in Fig. 1).

The protein of IL-22R1 also expressed in the cytoplasm and distributed in the epithelium, mesenchyma, submucosal glands, vascular endothelium, and inflammatory cells of normal tissues. Meanwhile, the expression of IL-22R1 was mainly distributed in tumor cells of LSCC. The expression of IL-22R1 in LSCC was significantly higher than controls (*P* < .001) (shown in Fig. 1).

**Figure 2:**
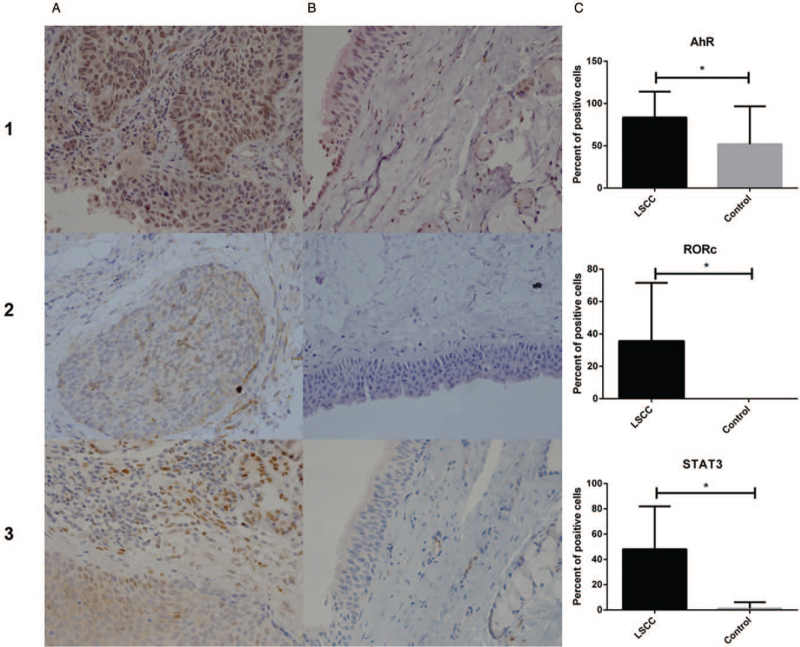
Immunohistochemical staining (A: LSCC; B: Controls) and the percentage of positive cells (C) of AhR (1), RORc (2), and STAT3 (3). Images acquired by optical microscope 400×. ^∗^*P* < .01; LSCC = laryngeal squamous cell carcinoma.

The protein of AhR mainly located in the nucleus and expressed in the epithelium, glands, and the inflammatory cells in the mesenchyme of control tissues. The expression of AhR was predominately distributed in the nucleus of tumor cells in LSCC. The expression of AhR was significantly higher in LSCC than in controls (*P* = .0015) (shown in Fig. 2).

RORc was not expressed in the control tissues, while weakly distributed in the tumor cells of LSCC. The expression of RORc was significantly different between LSCC and controls (*P* = .004) (shown in Fig. 2).

Protein of STAT3 located in the cell nucleus and mainly distributed in the epithelial cells, vascular endothelium, and glands. The expression of STAT3 was primarily presented in the tumor cell nucleus of LSCC. The expression of STAT3 in LSCC was significantly higher than that of controls (*P* = .001) (shown in Fig. 2).

### 3.3 Correlation between IL-22 and IL-22 receptor 1 expression and clinicopathological characteristics

The correlation between the expression of IL-22 and IL-22R1 and clinicopathological characteristics is summarized in Table 2. The expression of IL-22 and IL-22R1 was significantly related to lymph node metastasis (*P* = .002, *P* = .009) and clinical stage of LSCC (*P* = .001, *P* = .037). But the expression of IL-22 and IL-22R1 had weak correlation with patient gender (*P* = .662, *P* > .99), age (*P* = .153, *P* = .565), tumor location (*P* = .374, *P* > .99), T stage (*P* = .204, *P* = .140), and pathologic differentiation (*P* = .061, *P* = .626).

**Figure 3:**
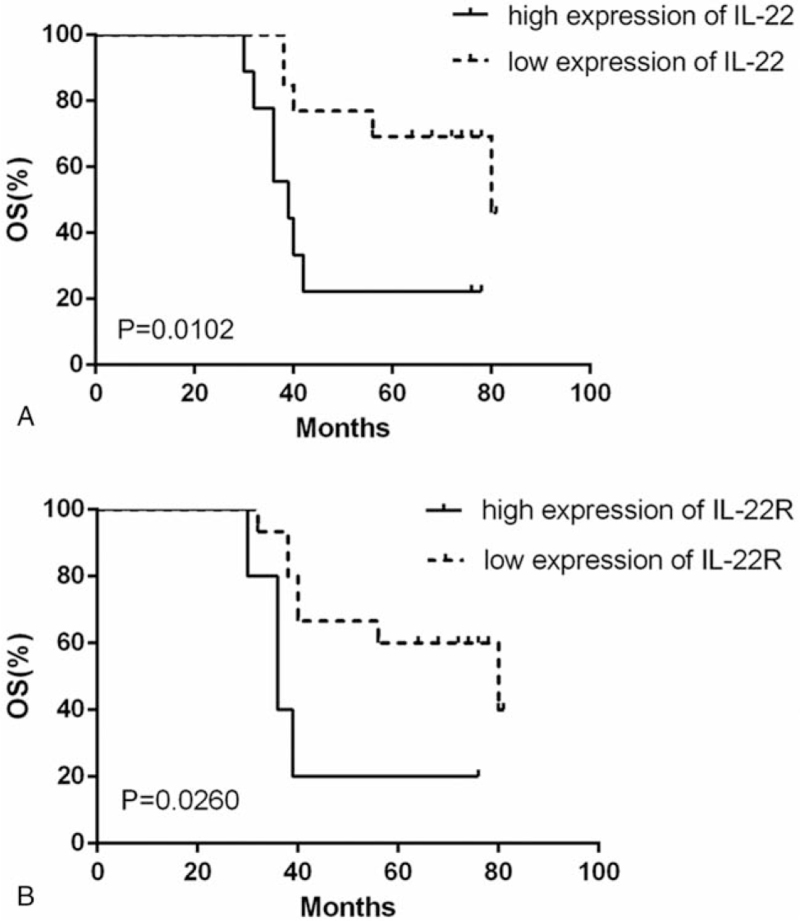
Kaplan–Meier survival curve showing overall survival based on IL-22 (A) and IL22R (B) expression. IL-22 = interlerukin-22, IL-22R = interleukin 22 receptor.

A Kaplan–Meier survival curve was used to evaluate overall survival of LSCC patients based on IL-22 expression (shown in Fig. 3). Patients with high expression of IL-22 had a shorter overall survival (median 39 months) than patients with low expression (median 80 months) (long rank test, *P* = .0102). The survival curve also showed a significant correlation between IL-22R1 expression and overall survival (shown in Fig. 3). Patients with elevated expression of IL-22R1 (median 36 months) had a shorter overall survival than patients with low expression (median 80 months) (long rank test, *P* = .026).

### 3.4 Expression of genes

**Figure 4:**
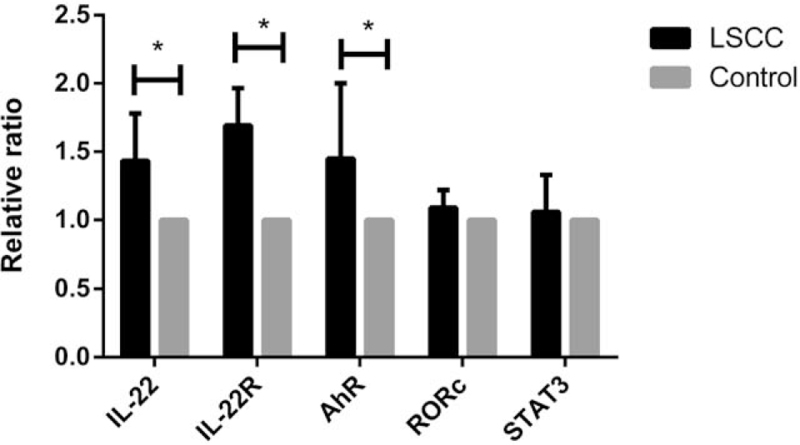
Relative ratio of IL-22 associated gene mRNA expression in LSCC (^∗^*P* < .05). IL-22 = interlerukin-22, LSCC = laryngeal squamous cell carcinoma.

The expression of mRNA of IL-22, IL-22R1, and correlative genes including AhR, RORc, and STAT3 was analyzed according to results of real time PCR. The expression of mRNA of IL-22, IL-22R1, and AhR in LSCC was significantly higher than that of controls (*P* = .012, .007, .028). But the expression of RORc and STAT3 had no significant difference between LSCC and controls (*P* = .348, .694) (shown in Fig. 4).

## 4 Discussion

LSCC is the solid tumor that has extensive interaction with the surrounding tumor microenvironment.^[11]^ The surrounding stroma and cellular infiltration compose the tumor microenvironment. Immune cells, both of the innate and adaptive systems, as well as other cytokines and signaling molecules, also contribute to the tumor microenvironment.^[12]^ The increasing evidence have suggested that innate and adaptive immune mediators in the tumor microenvironment play an important role in tumor progression of laryngeal carcinomas, such as IL-33, tumor necrosis factor superfamily member 13, neutrophil infiltration, and Treg cells.^[13–15]^

At present, IL-22 is well known as a cytokine secreted by lymphoid cells. The main source of IL-22 during steady-state is lymphoid cells (ILC3), besides Th17 and Th22 cells are almost the most source of IL-22 when the T cell receptor engages its cognate antigen during infections and diseases. Similar to ILC3, the degree of IL-22 produced by Th22 cells is cooperatively regulated by RORγt and AhR regulatory signal pathways.^[16]^ Stimulation of Th17 cells with AhR ligands results in preferential production of IL-22 and diminished release of IL-17.^[17]^ The important functions of IL-22 in host defense are involved in injury against, antimicrobial defense barrier and promote regeneration.^[4]^ Furthermore, IL-22 contributes to the pathogenesis of various malignant diseases. It has been reported that high level of IL-22 receptor by tumors as well as vast of IL-22 by lymphocytes surrounding tumor cells were expressed in various organs including lungs, liver, stomach, and colon.^[5]^ But few studies investigated the pathogenesis role of IL-22 in the LSCC. Recent research has suggested that the expression of Th17 cells, Th17 cytokine, and IL-17 represented a high level in laryngeal carcinoma.^[18–19]^ It has been shown that the expression of IL-22 was significantly up-regulated in the serum of patients with laryngeal carcinoma.^[19]^ The purpose of the present study was to elucidate the expression of IL-22, IL-22R, and correlative factors in LSCC and to explore the relationship of these factors in clinicopathological characteristics and prognosis.

IL-22 has been demonstrated the function of promoting tumor progression through mediating the target expression of IL22R in stem cells, and inducing the proliferation to enhance tumor growth. It has been demonstrated that IL-22 and IL-22R were overexpressed in colon cancer microenvironment. This contributes to the tumor growth, inhibition of apoptosis, and promotion of metastasis through STAT3 activation. Previous study showed increased serum levels of IL-22 in patients with non-small cell lung carcinoma and IL-22Ra1 expression up-regulated in tumor cells. Moreover, it has been shown that IL-22 promotes human hepatocellular carcinoma via activation of STAT3. IL-22R1 is restricted in cell lineage of a nonhematopoietic origin. The restricted distribution of the IL-22R1 governs the functions of IL-22. IL-22R1 expression is mostly resident in the epithelial cells of the skin, lung, and gut including hepatocytes, and kidney.^[4]^ By immunochemical staining and real-time quantitative PCR, we detected the expression of IL-22 and IL-22R1 in LSCC tissue. Our results showed that the expression of IL-22 and IL-22R1 was significantly higher in LSCC than in controls, not only in mRNA level but also at the protein level. Laryngeal mucosa consists of the epithelium of the respiratory and digestive tract. This study suggested IL-22 and IL-22R1 play an important role in the pathogenesis of laryngeal carcinoma.

The extracellular matrix (ECM) is a structure of non-cellular network surrounding cells which provides physical and biochemical support. Deregulated and disorganized ECM stimulates malignant cells transformation into cancer. Increased production of collagen, laminin, and elastin in ECM also has a strong impact on cancer progression.^[20]^ In the present study, it has been observed that the positive staining of IL-22 accumulated in tumor cells, mesenchyme and fibrous tissues around cancer nests of LSCC. While IL-22R1 mainly distributed in tumor cells of LSCC. Kaplan–Meier analysis indicated that high expression of IL-22 and IL-22R1 was relevant to the poor prognosis of LSCC patients. This study provided evidence that IL-22 and IL22R1 were elevated in the microenvironment of LSCC. It was associated with lymph node involvement and poor prognosis. IL-22 and IL-22R1 maybe as potential markers of prognostic for LSCC. This was in consistent with the results of studies of gastric cancer and pancreatic cancer.^[7]^

The AhR is a ligand-dependent transcription factor that plays a critical role in maintaining homeostasis and in triggering pathology by modulating the biological responses of critical cells at the barrier and mucosal interfaces. AhR is critical to ILC3-derived IL-22 production, either as a direct regulator of IL-22 gene expression or a regulator of ILC3 and Th17. The transcription factor RORγt is constitutively expressed and also required for IL-22 production.^[21]^ In this study, we have investigated the expression of AhR and RORc in LSCC, and revealed that the expression of AhR was significantly up-regulated, however, RORc presented down-regulated. Distribution of AhR is normally in the cytoplasm of cells, but it translocates to the nucleus upon ligand binding. Our results showed the expression of AhR predominately located in the nucleus of tumor cells in LSCC. Results of real time PCR suggested that mRNA expression of AhR was significantly up-regulated in LSCC, but RORc has no such phenomenon. So AhR is particularly relevant to IL-22 function in LSCC.

Binding of IL-22 to its receptor triggers a cascade of downstream signaling pathways. STAT3 phosphorylation appears to be the primary mediator of IL-22 signaling. STAT3 signaling plays an important role during cancer progression. It has been proved that IL-22 may directly promote tumor growth by engaging STAT3 signaling in tumor cells. The STAT3 phosphorylation induced by IL-22 was observed in human colonic cancer, hepatocellular carcinoma, lung cancer, and oral squamous cell carcinoma.^[5,9]^ In the immunohistochemical experiment, the expression of STAT3 was primarily observed in the nucleus of tumor cells and significantly up-regulated in LSCC. But there was no significant difference of mRNA expression of STAT3 between LSCC and controls. Further research is needed to bring to light the role of IL-22 in activating STAT3 and subsequent cytokines in LSCC in vivo. The biological effect in LSCC of STAT molecules mediated by IL-22, such as STAT1, STAT5, needs to be addressed in the future.

## 5 Conclusions

**Figure 5:**
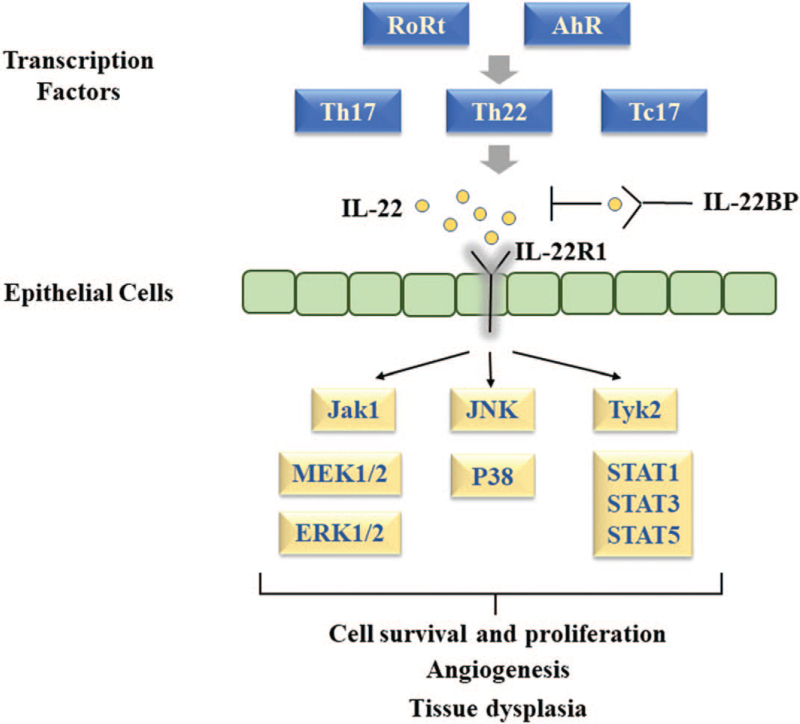
The function of IL-22/IL-22R in cancer cells. IL-22 = interlerukin-22, IL-22R = interleukin 22 receptor.

The host response mediated by immune cells in the tumor microenvironment makes a significant contribution to the tumor development. It is beneficial to identify cytokines as biomarkers of malignant transformation in guiding diagnosis and targeted therapy. IL-22 regulates a globally altered gene expression profile in tumor cells, including signaling pathways, cell proliferation, and migration (Fig. 5). In this study we investigated the expression of IL-22, IL-22R1, AhR, RORc, and STAT3 in LSCC. Our results showed that higher expression of IL-22 and IL-22R1 was associated with lymph node involvement and a poor prognosis in LSCC. AhR was the primary transcription factor and STAT3 was the key signaling mediator on the function of IL-22 in LSCC. However, there were some limitations in this study, such as small sample size, without validation study in cell lines and in vivo. Further research is required to elucidate the mechanism of IL-22 effecting directly and indirectly on tumor cells in LSCC. IL-22 may be a therapeutic target for laryngeal carcinoma.

## Author contributions

**Conceptualization:** Xin Wang.

**Data analysis:** WenJun Ji, Jing Li, TianTian Zhang.

**Data curation:** Wenjun Ji, Jing Li.

**Experimental detection:** WenJun Ji, Jing Li, DongSheng Gao.

**Formal analysis:** Wenjun Ji, Jing Li.

**Funding acquisition:** Xin Wang.

**Investigation:** Wenjun Ji, Jing Li, Dongsheng Gao.

**Software:** Dongsheng Gao.

**Supervision:** Xin Wang.

**Writing – original draft:** Wenjun Ji.

**Writing – review & editing:** Xin Wang.
